# Predominance of *Clostridium difficile* Ribotypes 017 and 078 among Toxigenic Clinical Isolates in Southern Taiwan

**DOI:** 10.1371/journal.pone.0166159

**Published:** 2016-11-18

**Authors:** Yuan-Pin Hung, I-Hsiu Huang, Hsiao-Ju Lin, Bo-Yang Tsai, Hsiao-Chieh Liu, Hsiu-Chuan Liu, Jen-Chieh Lee, Yi-Hui Wu, Pei-Jane Tsai, Wen-Chien Ko

**Affiliations:** 1 Department of Internal Medicine, Tainan Hospital, Ministry of Health & Welfare, Tainan, Taiwan; 2 Department of Experiment and Diagnosis, Tainan Hospital, Ministry of Health & Welfare, Tainan, Taiwan; 3 Department of Internal Medicine, National Cheng Kung University Hospital, Tainan, Taiwan; 4 Center of Infection Control, National Cheng Kung University Hospital, Tainan, Taiwan; 5 Graduate Institute of Clinical Medicine, National Cheng Kung University Hospital, Tainan, Taiwan; 6 Department of Medicine, College of Medicine, National Cheng Kung University, Tainan, Taiwan; 7 Department of Microbiology and Immunology, College of Medicine, National Cheng Kung University, Tainan, Taiwan; 8 Department of Medical Laboratory Science and Biotechnology, College of Medicine, National Cheng Kung University, Tainan, Taiwan; 9 Department of Internal Medicine, E-da Hospital, Kaohsiung, Taiwan; 10 Center of Infectious Disease and Signaling Research, National Cheng Kung University, Tainan, Taiwan; Cleveland Clinic, UNITED STATES

## Abstract

Ribotypes and toxin genotypes of clinical *C*. *difficile* isolates in Taiwan are rarely reported. A prospective surveillance study from January 2011 to January 2013 was conducted at the medical wards of a district hospital in southern Taiwan. Of the first toxigenic isolates from 120 patients, 68 (56.7%) of 120 isolates possessed both *tcdA* and *tcdB*. Of 52 (43.3%) with *tcdB* and truncated *tcdA* (*tcdA*-/*tcdB*+), all were ribotype 017 and none had binary toxin or *tcdC* deletion. Eighteen (15%) toxigenic isolates harbored binary toxins (*cdtA* and *cdtB*) and all had *tcdC* deletion, including Δ39 (C184T) deletion (14 isolates), Δ18 in-frame deletion (3 isolates), and Δ18 (Δ117A) deletion (1 isolate). Eleven of 14 isolates with Δ39 (C184T) deletion belonged to the ribotype 078 family, including ribotype 127 (6 isolates), ribotype 126 (4 isolates), and ribotype 078 (1 isolate). Among 8 patients with consecutive *C*. *difficile* isolates, these isolates from 6 (75%) patients were identical, irrespective of the presence or absence of diarrhea, suggestive of persistent fecal carriage or colonization. In conclusion in southern Taiwan, ribotype 017 isolates with a *tcdA*-/*tcdB*+ genotype were not uncommon and of *C*. *difficile* isolates with binary toxin, the ribotype 078 family was predominant.

## Introduction

*Clostridium difficile* is the leading cause of nosocomial diarrhea with an increase in the incidence of sporadic outbreaks causing severe and fatal infections since the beginning of the century [1]. Most alarming is the outbreak of *C*. *difficile* infections (CDIs) in Quebec, Canada in 2003. During the outbreak involving 1,703 patients, CDI was the attributable cause of death in 117 (6.9%) cases and a contributing factor in additional 127 (7.5%) deaths [2]. The hypervirulent strain had been assigned as the North American pulse-field type 1 (NAP1), restriction endonuclease analysis (REA) group BI, and polymerase chain reaction (PCR) ribotype 027 (sometimes referred to as BI/NAP1/027). Three bacterial factors have been found in the epidemic *C*. *difficile* strain, including *in vitro* increased production of toxin A and B, fluoroquinolone resistance, and production of binary toxin [1]. Toxin A and B are transcribed from a pathogenicity locus comprised of five genes: *tcdA* (toxin A), *tcdB* (toxin B), and three regulatory genes. One of the latter, *tcdC*, is a negative regulator of toxin production. Binary toxin is transcribed from *cdtA* and *cdtB* [1].

Changing *C*. *difficile* epidemiology is noted worldwide. In the European Study Group of *C*. *difficile* (ESGCD) between 2002 and 2005, major toxigenic ribotypes were 001 (13%), 014 (9%), 002, 012, 017, 020, and 027 (each about 6%), with the CDI incidence ranging from 0.13 to 7.1 cases per 10,000 patient-days in different countries [3,4]. In Japan, a shift of the predominant ribotype, from PCR ribotype a in 2000 (15/33, 45%) to PCR ribotype f (type smz) in 2004 (18/28, 64%), was noted in a teaching hospital between 2000 and 2004 [5,6]. In Korea, *tcdA*-/*tcdB*+ *C*. *difficile* strains accounted for <7% in 2002, but increased to 13.2% in 2003 and 50.3% in 2004. In Taiwan ribotype 027, 078, or 001 isolates were not reported [7–9], until 2012 when the first case of CDI due to ribotype 027 was reported [10]. Nevertheless the information regarding toxin genotype and ribotype distribution in Taiwan remains scarce.

Genetic relationship of the *C*. *difficile* isolates causing colonization, infection, or recurrence in the same individual remains variable in several studies. Among 20 recurrent cases, Oka found 16 (80%) cases were identical between the strains at initial infection and at recurrence [11]. Nevertheless Barbut *et al*. reported that of the strains from 93 hospitalized patients with recurrent CDI between 1994 and 1997, 48.4% of clinical recurrences were caused by different strains compared with initial strains [12]. However, the question of whether the initial colonized *C*. *difficile* strain was the same as or different from the strain causing subsequent infection was not answered.

In our previously published data, we reported the clinical impact and risk factor of *C*. *difficile* colonization and infection in a prospective study from January 2011 to June 2012 [8,13,14]. We analyzed the clinical *C*. *difficile* isolates during the study period and found the first hypervirulent *C*. *difficile* ribotype 126 strain in Taiwan [15]. We further extended the prospective study to January 2013. In this study we aimed to investigate the toxin gene content and ribotype distribution of *C*. *difficile* isolates with *tcdC* deletion collected from previous studies [13–15].

## Materials and Methods

### Study design

We collected clinical *C*. *difficile* isolates from stool culture of the prospective study from January 2011 to June 2012 as described before [8,13,14]. Briefly a prospective investigation was conducted in the medical wards of the Tainan Hospital, Ministry of Health and Welfare, a district hospital in southern Taiwan. The prospective clinical study was further extended to January 2013. The study was approved by the institutional review board of the Tainan Hospital, Ministry of Health and Welfare, and written informed consents were obtained from enrolled patients. Patients with age of at least 20 years old, and admitting to medical wards with expected hospital stays of at least 5 days were included. Exclusion criteria were patients with previous metronidazole or oral vancomycin therapy within three months, colectomy, or CDI at admission [16–18]. We retrieved demographic information, laboratory data, medication history, and underlying disease from medical records. Stool samples from the patients included in the study from January 2011 to January 2013 were sent for *C*. *difficile* culture. Stool samples were plated on cycloserine–cefoxitin-fructose agar (CCFA) under anaerobic conditions. *C*. *difficile* colonization (CdC) is defined as an asymptomatic patient with the presence of *C*. *difficile* in stool and CDI as a patient with diarrhea and the detection of toxigenic *C*. *difficile* in stool. Recurrence was defined as the resurgence of clinical symptoms after cessation of antimicrobial therapy, at least 10 days after the first episode [12].

### Bacterial strains

*C*. *difficile* CCUG4938T (ribotype 001 with wild type *tcdC*, toxinotype 0, purchased from the Culture Collections of the University of Goteborg, Sweden), a ribotype 078 strain (with a 39-bp deletion in *tcdC*, provided by Prof. EJ Kuijper at Leiden University Medical Center, the Netherlands), and ATCC BAA1805 (ribotype 027 with an 18-bp deletion of *tcdC*, purchased from American Type Culture Collection, USA), ribotype 106 and ribotype 001/072 strain (provided by Prof. Ellie JC Glodstein at UCLA, USA), were used as reference strains.

### Genomic DNA

*C*. *difficile* strains were grown anaerobically in Brain Heart Infusion broth (Becton, Dickinson and Company) with 5 mg/ml yeast extract (MO BIO Laboratories, Inc.) and 0.1% L-cysteine (AMRESCO^®^) at 37°C for two days. After harvesting the bacteria, *C*. *difficile* genomic DNA was extracted with a genomic DNA mini kit (Geneaid, Ltd, Taiwan).

### Detection of toxin genes

The extracted DNA was amplified for the 16s rDNA, *tcdA*, *tcdB*, *cdtA*, *cdtB*, and *tcdC* genes of *C*. *difficile* in a single multiplex PCR, as described in [19]. The strains containing a truncated *tcdC* profile were further examined through *tcdC* sequencing, as previously described in [20]. Sequencing was performed by Mission Biotech Co., Ltd. Amplification was performed with a BigDye terminator 3.1 kit (Applied Biosystems) according to the manufacturer’s instructions. Capillary sequence analysis was also performed by Mission Biotech, Taiwan with an ABI 3730xl DNA sequencer (Applied Biosystems).

### Antimicrobial susceptibility

Overnight cultures of *C*. *difficile* strains were inoculated onto Brucella agar (Oxoid) plates containing vitamin K1 (0.5 mg/L), haemin (5 mg/L) and 5% defibrinated sheep red blood cells. Minimum inhibitory concentrations (MICs) of moxifloxacin (MX), metronidazole (MZ), and vancomycin (VA) were evaluated by Etest (AB Biodisk, Solna, Sweden). Quality control strains included *Bacteroides fragilis* ATCC25285, *Bacteroides thetaiotaomicron* ATCC 29741, and *C*. *difficile* ATCC700057. The breakpoint used for three tested antibiotics was 8 mg/L, 32 mg/L and 16 mg/L, respectively, in accordance with the guideline established by the Clinical and Laboratory Standards Institute (CLSI).

Sequence analysis of *gyrA* and *gyrB* was performed as previously described [21]. Briefly, the DNA region was amplified using the primer pairs, gyrA1–gyrA2 for *gyr*A and gyrB1–gyrB2 for *gyr*B. PCR products were purified and sequenced. Pairwise alignments of DNA sequences were carried out using the BLAST server of the National Center for Biotechnology Information.

### PCR ribotyping

The isolates containing *tcdB* and determined as toxigenic strains were further examined by polymerase chain reaction (PCR) ribotyping. The ribotyping method and PCR primers used were as described previously [22]. After PCR amplification, the samples were concentrated using the Gel/PCR DNA Fragments Extraction Kit (Geneaid, Ltd, Taiwan) and separated by the QIAxcel capillary electrophoresis system (Qiagen, Hilden, Germany) using the “OM500” method and QX Alignment Marker 15 bp/3 kb (Qiagen, Hilden, Germany).

### Repetitive sequence-based polymerase chain reaction (Rep-PCR)

The identity of the consecutive isolates collected beyond 28 days from the same patient was determined by Rep-PCR. Rep-PCR was performed as described by Versalovic *et al*. [23], and the PCR products were analyzed using the QIAxcel system.

### Statistical analysis

Statistical analyses were performed by statistical software (SPSS, version 13.0). Continuous data were expressed as the means ± standard deviations. The χ^2^ test or Fisher’s test was used to compare categorical variables, and Student’s t-test was used to compare continuous variables. A two-tailed *P* value of less than 0.05 was considered to be statistically significant.

## Results

### Toxin gene content distribution of clinical *C*. *difficile* isolates

**Fig 1:**
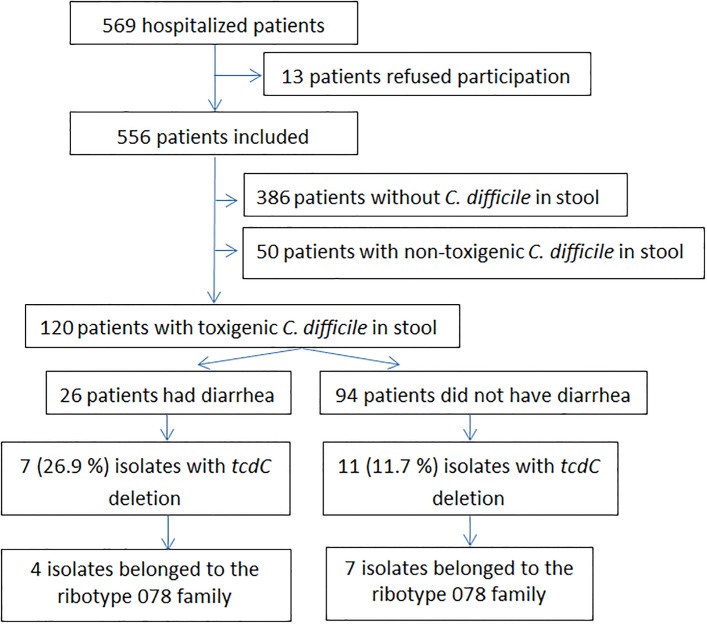
Flowchart of the patients enrolled in this study.

Of 569 hospitalized patients, 556 fulfilling the inclusion criteria were enrolled. *C*. *difficile* was found in 170 patients. Overall 120 (70.6%) patients had toxigenic *C*. *difficile* isolates harvested from stool and 50 (29.4%) harboured non-toxigenic *C*. *difficile* isolates (Fig 1). Of 120 patients, 26 (21.7%) developed diarrhea and were regarded as having CDI while 94 patients had toxigenic *C*. *difficile* colonization (tCdC). Of 120 toxigenic *C*. *difficile* isolates, 68 (56.7%) possessed both *tcdA* and *tcdB* and 52 (43.3%) had *tcdB* and truncated *tcdA* (Table 1). Among 68 *tcdA*+/*tcdB+* isolates, 18 (26.4%) harbored binary toxin (*cdtA* and *cdtB*) and *tcdC* deletion, including Δ39 (C184T) deletion (14 isolates), Δ18 in-frame deletion (3 isolates), and Δ18 (Δ117A) deletion (1 isolate). Of 52 *tcdA-/tcdB+* isolates, none had binary toxin or *tcdC* deletion.

**Table 1:** Toxin gene contents of 120 toxigenic clinical *Clostridium difficile* isolates.

Toxin genes, isolate No. (%)	CDT	*tcdC* pattern	Isolate No. (%)
*tcdA*+/*tcdB*+, 68 (56.7)	CDT+	Δ18 bp, in-frame	3 (2.5)
	CDT+	Δ18 bp, Δ117A	1 (0.8)
	CDT+	Δ39 bp, C184T	14 (11.7)
	CDT-	Wild type	50 (41.7)
*tcdA*-/*tcdB*+, 52 (43.3)	CDT-	Wild type	52 (43.3)

CDT = *C*. *difficile* binary toxin; bp = base-pair.

### Genetic relationship of consecutive toxigenic *C*. *difficile* isolates from the same individual

**Fig 2:**
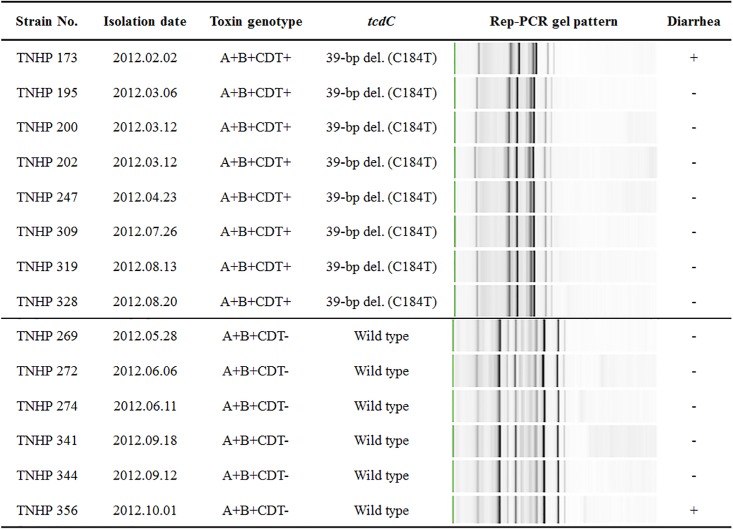
Molecular characteristics of consecutive fecal isolates of *Clostridium difficile* obtained from two patients, TNHP 173~TNHP 328 (ribotype 126) and TNHP 269~ TNHP 356 (ribotype 106), respectively. A = *tcdA*; B = *tcdB*; CDT = binary toxin; bp = base-pair; del. = deletion; Rep-PCR = repetitive sequence-based polymerase chain reaction.

Genetic relationship of consecutive toxigenic isolates from the same individual collected beyond 28 days was examined by Rep-PCR (Table 2). Of 8 patients, consecutive isolates were identical in 6 (75.0%) patients, irrespective of the presence or absence of diarrhea. Genetic similarity among consecutive fecal *C*. *difficile* isolates obtained from 2 patients was illustrated (Fig 2).

**Table 2:** The toxin gene content and ribotype of consecutive toxigenic *Clostridium difficile* isolates.

Patient No.	Follow-up period	Clinical condition	Strain shift*	Toxin gene content	Ribotype (RT)
1	2011/11/9-2012/2/15	C→D→C	Yes	A+B+CDT-→ A-B+CDT-	unknown→RT 017
2	2012/4/17-2012/11/30	C→D→D	Yes	A+B+CDT-→ A+B+CDT- → A+B+CDT-	RT 106→RT 001/072 →unknown
3	2012/2/2-2012/8/20	D→C	No	A+B+CDT+	RT 126
4	2012/3/16-2012/4/16	D→C→D	No	A+B+CDT-	RT 106
5	2012/4/20-2012/10/11	C→D	No	A-B+CDT-	RT 017
6	2012/5/28-2012/10/1	C→D	No	A+B+CDT-	RT 106
7	2012/6/20-2012/7/18	C→D	No	A+B+CDT-	unknown
8	2012/8/9-2012/9/7	C→D→C→D	No	A+B+CDT-	unknown

A = *tcdA*; B = *tcdB*; C = colonization (*C*. *difficile* colonization); CDT = *cdtA/cdtB*; D = disease (*C*. *difficile* infection).

* Indicates the detection of other rep-PCR profiles.

### Ribotype and antimicrobial susceptibility of *C*. *difficile* isolates

**Fig 3:**
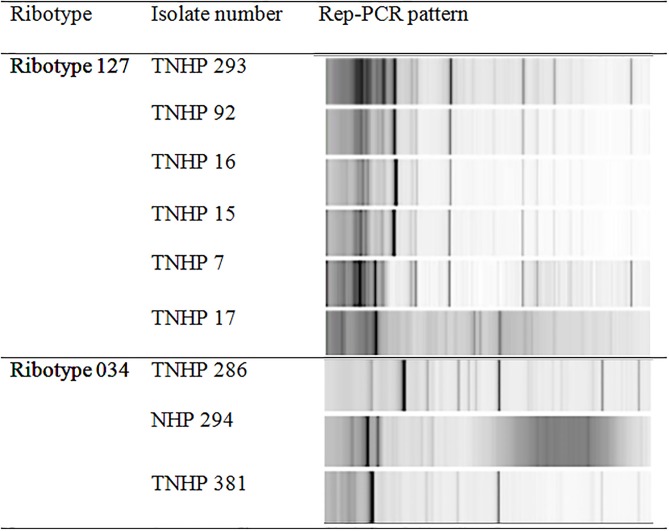
Repetitive sequence-based polymerase chain reaction (Rep-PCR) gel patterns of 6 toxigenic *Clostridium difficile* isolates of ribotype 127 (3 subtypes) and ribotype 034 isolates (3 subtypes).

The ribotypes, antimicrobial susceptibility, and partial sequences of *gyrA/gyrB* of 18 toxigenic isolates with binary toxin and *tcdC* deletion were investigated (Table 3). Eleven (61.1%) isolates belonged to the ribotype 078 family, including ribotype 078 (1 isolate), ribotype 126 (4 isolates), and ribotype 127 (6 isolates) (S1 Fig). Of the 52 isolates with *tcdA-/tcdB+* genotype, all were identified to be ribotype 017. Prof. M Wilcox at the Leeds Teaching Hospitals NHS Trust confirmed ribotyping of the above clinical isolates. For genetic relationship studied using Rep-PCR, 4 isolates of ribotype 126 were found to be identical as reported previously [15], and 6 isolates of ribotype 127 exhibited 3 distinct subtypes with a predominant Rep-PCR subtype (4 isolates). However, 3 isolates of ribotype 034 were genetically different (Fig 3). All 18 toxigenic isolates with binary toxin were susceptible to metronidazole and vancomycin *in vitro*, and of 10 (55.6%) moxifloxacin-resistant isolates (MIC >32 mg/L), 9 had *gyrA* mutation (Thr82Ile) and 1 had *gyrB* mutation (Asp426Asn).

**Table 3:** Ribotypes, *gyrA* and *gyrB* mutations, and antimicrobial susceptibility of 18 *Clostridium difficile* isolates with binary toxin.

Isolate No.	Ribotype (RT)	*gyrA* mutation	*gyrB* mutation	MIC, mg/L		
				MX	MZ	VA
2	RT 328	-	-	0.5	0.032	0.5
286	RT 034	-	-	0.5	0.064	0.5
294		-	Asp426Asn	>32	0.125	0.5
381		-	-	0.5	0.032	0.25
80	RT 078	-	-	0.25	0.032	0.38
61	RT 126	Thr82Ile	-	>32	0.032	0.25
173		Thr82Ile	-	>32	0.032	0.5
203		-	-	0.5	0.032	0.75
264		-	-	0.5	0.047	0.75
7	RT 127	Thr82Ile	-	>32	0.032	0.38
15		Thr82Ile	-	>32	0.032	0.38
16		Thr82Ile	-	>32	0.032	<0.016
17		Thr82Ile	-	>32	<0.016	0.38
92		-	-	0.38	0.032	0.38
293		Thr82Ile	-	>32	0.023	0.38
9	Unknown	Thr82Ile	-	>32	0.032	0.38
13		Thr82Ile	-	>32	0.032	0.38
14		-	-	0.1	0.047	<0.016

MX: moxifloxacin; MZ: metronidazole; VA: vancomycin.

### Clinical characteristics of patients with *C*. *difficile* ribotype 078 family

Of 26 isolates causing CDI, 7 (26.9%) had binary toxin and 4 (15.4%) belonged to the ribotype 078 family. In contrast, 11 (11.7%) of 94 tCdC isolates had binary toxin and 7 (7.4%) isolates belonged to the ribotype 078 family (S1 Table). Clinical characteristics of 26 patients with CDI and 94 patients with tCdC were compared (Table 4). Patients with CDI were less likely to be nursing home residents (57.7% *vs*. 77.7%; *P* = 0.049), and more likely to have nasogastric tube (77.3% *vs*. 46.8%, *P* = 0.016) or diabetes mellitus (61.5% *vs*. 39.4%, *P* = 0.049). However, there were no differences in underlying disease, laboratory findings, ribotype distribution, and moxifloxacin resistance in two groups (Tables 4 and 5).

**Table 4:** Clinical characteristics of 120 patients with toxigenic *Clostridium difficile* in stool, stratified by the presence (*C*. *difficile* infection, CDI) or absence (*C*. *difficile* colonization, CdC) of diarrhea.

Characteristics	CDI, n = 26	CdC, n = 94	*P* values
Male	17 (65.4)	51 (54.8)	0.377
Age, years	70.5 ± 12.7	73.6 ± 13.5	0.274
Body weight, kg	50.2 ± 12.1	50.6 ± 11.2	0.404
Nursing home residents	15 (57.7)	73 (77.7)	0.049
Recent hospitalization within three months	9 (34.6)	28 (29.8)	0.638
Nasogastric tube use	17 (77.3)	44 (46.8)	0.016
Prior exposure to antibiotic*	7 (30.4)	23 (24.5)	0.598
Prior exposure to proton pump inhibitor*	2 (8.7)	5 (5.3)	0.622
Underlying medical diseases			
Hypertension	10 (38.5)	45 (47.9)	0.506
Diabetes mellitus	16 (61.5)	37 (39.4)	0.049
Old stroke	11 (42.3)	28 (29.8)	0.244
Chronic kidney disease (Ccr <60 ml/min)	3 (11.5)	19 (20.2)	0.400
On hemodialysis	1 (3.8)	8 (8.5)	0.682
Malignancy	4 (15.4)	9 (9.6)	0.475
Laboratory data, mean ± standard deviation			
White blood count, 10^3^/mm^3^	11.4 ± 5.8	11.5 ± 5.9	0.977
Neutrophils, %	75.5 ± 12.6	76.1 ± 15.1	0.856
Hemoglobin, g/dL	10.4 ± 2.3	11.92 ± 2.1	0.117
Platelet, 10^3^/mm^3^	253.7 ± 105.6	219.2 ± 107.5	0.152
Alanine aminotransferase, U/L	22.5 ± 24.3	39.9 ± 70.5	0.058
Creatinine, mg/dL	1.5 ± 1.0	2.6 ± 2.8	0.214

Data are no. (%) of patients, unless otherwise indicated; Ccr = creatinine clearance.

*Medication within three months before admission.

**Table 5:** Ribotype (RT), binary toxin, and *gyrA/B* mutation of initial toxigenic *Clostridium difficile* isolates from 120 patients, stratified by the presence (*C*. *difficile* infection, CDI) or absence (*C*. *difficile* colonization, CdC) of diarrhea.

Characteristics	CDI, n = 26	CdC, n = 94	*P* values
Binary toxin	7 (26.9)	11 (11.7)	0.067
RT 078 family	4 (15.4)	7 (7.4)	0.250
RT 078	0	1	
RT 126	3	1	
RT 127	1	5	
RT 034	2	1	
RT 328	0	1	
Unknown ribotype	1	2	
*gyr* mutation			
*gyrA* mutation	5 (19.2)	9 (9.6)	0.181
*gyrB* mutation	6 (23.1)	8 (8.5)	0.077

Data are no. (%) of patients, unless otherwise indicated.

We assessed the seasonal distribution of toxigenic *C*. *difficile* isolates stratified by binary toxin and ribotype 078 family (S2 Fig). More *C*. *difficile* isolates with binary toxin, particularly those belonging to the ribotype 078 family, were harvested at the first and second quarters of 2011 and 2012.

## Discussion

In the present study, the ribotype 078 family was dominant among toxigenic *C*. *difficile* isolates with binary toxin and *tcdC* deletion in Taiwan. Moreover the ribotype 078 family in Taiwan often displayed a high level of moxifloxacin resistance. Several retrospective studies in Taiwan investigated the prevalence of hypervirulent *C*. *difficile* strains, such as ribotype 027 or 078, but no hypervirulent isolates had been discovered before 2012 [7–9]. To our knowledge, this is the first study describing ribotype and toxin genotype distribution of clinical *C*. *difficile* isolates in Taiwan. Our study revealed that among the isolates with binary toxin, more than half belonged to the ribotype 078 family, including ribotypes 078, 126, and 127. The result was unique in that ribotype 078 has been reported from Korea and China [24–26], but it not the dominant ribotype. Clinical impact and origin of the dominant ribotype 078 family among binary toxin producers in Taiwan warrant further evaluations.

In Hangzhou, China in 2013, the predominant *C*. *difficile* ribotypes in hospitalized cancer patients included 001, 017/1, and 017 [27]. At another hospital in Hunan, China between April 2009 and February 2010, the dominant ribotype was 017, followed by 046 and 012 [28]. Ribotypes 018, 017 and 001 were prevalent in Seoul, Korea from September 2008 to January 2010 [29]. In general, ribotype 017 isolates were commonly present in China and Korea, and here we found that this ribotype accounted for 43% of toxigenic isolates, suggestive of its widespread in Asia countries. An international surveillance of toxigenic *C*. *difficile* isolates is essential to disclose ribotype distribution and clinical significance of toxigenic *C*. *difficile* isolates in Asia.

Of the patients in our study, identical *C*. *difficile* isolates can be obtained from the same individuals with tCdC, CDI, or recurrent CDI. The result was similar to the study conducted by Oka *et al*., who noted 80% were identical between the strains at initial infection and at recurrence [11]. Moreover Kim *et al*. found relapse rates of ribotype 017 and 018 isolates were higher than those of other ribotypes (63.6% and 63.6% *vs*. 22.2%, respectively) [30]. The clinical impact of *C*. *difficile* ribotypes on CDI recurrence warrants more studies. However, in our study the same *C*. *difficile* isolate persisted from CdC to CDI or from CDI to CdC, suggestive of long-term carriage of identical *C*. *difficile* strains, irrespective of the initial presence or absence of diarrhea. Furthermore, the role of asymptomatic carriers in *C*. *difficile* transmission has been supported by a recent study in which active surveillance detection and isolation of *C*. *difficile* carriers was associated with a significant decrease in the incidence of healthcare-associated CDI [31]. Clinical significance and management of asymptomatic *C*. *difficile* carriers warrant more attention.

Patients with CDI were characterized as less likely to be nursing home residents, more likely to use nasogastric tube and to have diabetes mellitus, than those with CdC. Nasogastric tube use [32,33] and diabetes mellitus [34] had been regarded as risk factors for CDI before. It is notable that CDI in our study was inversely correlated with nursing home residing, though the prevalence of *C*. *difficile* colonization or infection among residents in long-term care facilities had been increasing in recent years due to advanced age, the recipients of multiple courses or longer duration of antibiotics, and previous CDI history [35,36].

There are some limitations in our study. Firstly, we only analyzed the ribotypes of toxigenic *C*. *difficile* isolates with binary toxin and *tcdC* deletion and *tcdA*-/*tcdB*+ isolates. Secondly, the *C*. *difficile* isolates studied were only obtained from a district hospital in southern Taiwan. Their representativeness is of course questionable. Thirdly, it is likely that the boundary of *C*. *difficile* colonization and infection is blurred. With longer followed-up periods, the isolates of tCdC and CDI will be exchangeable. For example, the isolates initially causing colonization can later be related to CDI, and vice versa. Finally, though a total of 13 patients were excluded from the study due to expected hospital stay for less than 5 days, recent metronidazole or vancomycin therapy, colectomy, or CDI at admission, the estimated number of toxigenic *C*. *difficile* isolates obtained from them was 2 or 3. Therefore, the exclusion of these patients will not change significantly our study results.

In conclusion, ribotype 017 isolates were not uncommon in toxigenic *C*. *difficile* isolates southern Taiwan and the ribotype 078 family predominated among clinical *C*. *difficile* isolates with binary toxin.

## Supporting Information

S1 Fig Gel patterns of different *Clostridium difficile* ribotypes. (TIF)

S2 Fig Quarterly distribution of the isolate numbers of *Clostridium difficile*, stratified by *tcdC* deletion and ribotype (RT) 078 family. (TIF)

S1 Table Clinical characteristics of 120 enrolled patients and ribotype and binary toxin in their fecal *Clostridium difficile* isolates. (XLS)
