# Supplementary figures and images for: Predominance of Clostridium difficile Ribotypes 017 and 078 among Toxigenic Clinical Isolates in Southern Taiwan
Source: PLoS One. 2016 Nov 18;11(11):e0166159. doi: 10.1371/journal.pone.0166159 (PMC5115699; doi:10.1371/journal.pone.0166159)

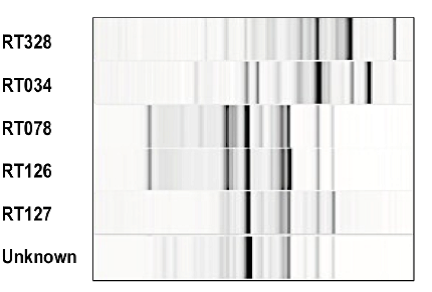

Supplement: S1 Fig — (TIF) [file pone.0166159.s001.tif]

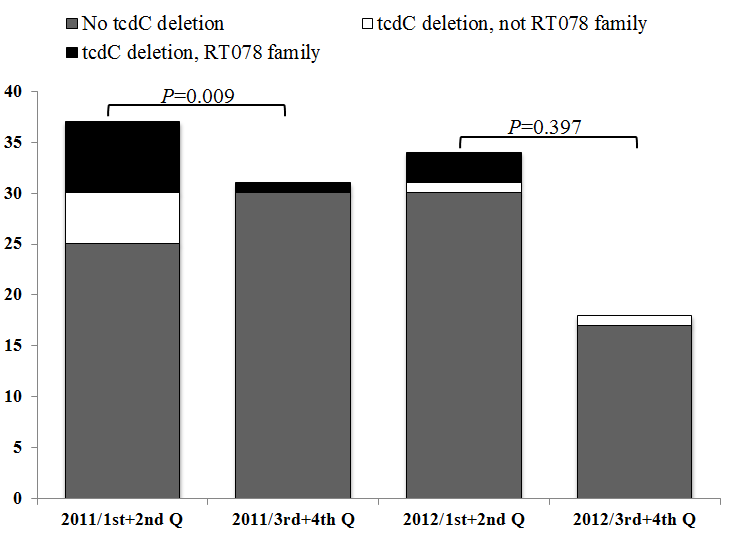

Supplement: S2 Fig — (TIF) [file pone.0166159.s002.tif]
